# Effects of *Clostridium butyricum*, Sodium Butyrate, and Butyric Acid Glycerides on the Reproductive Performance, Egg Quality, Intestinal Health, and Offspring Performance of Yellow-Feathered Breeder Hens

**DOI:** 10.3389/fmicb.2021.657542

**Published:** 2021-09-16

**Authors:** Yibing Wang, Yang Wang, Xiajing Lin, Zhongyong Gou, Qiuli Fan, Shouqun Jiang

**Affiliations:** ^1^State Key Laboratory of Livestock and Poultry Breeding, Key Laboratory of Animal Nutrition and Feed Science in South China, Ministry of Agricultural and Rural Affairs, Guangdong Provincial Key Laboratory of Animal Breeding and Nutrition, Institute of Animal Science, Guangdong Academy of Agricultural Sciences, Guangzhou, China; ^2^College of Animal Science and Technology, Qingdao Agricultural University, Qingdao, China

**Keywords:** reproductive performance, *Clostridium butyricum*, sodium butyrate, butyric acid glycerides, yellow-feathered breeder hens

## Abstract

Butyrate has been reported to promote the performance and growth of chickens. The specific roles and efficacy of different sources of butyrate remained unclear. Thus, the present study aimed to investigate and compare the effects of *Clostridium butyricum* (CB), sodium butyrate (SB), and butyric acid glycerides (tributyrin, BAG) on the reproductive performance, egg quality, intestinal health, and offspring performance of yellow-feathered breeder hens. A total of 300 Lingnan yellow-feathered breeder hens were assigned to five treatment groups: control (CL), 1×10^8^CFU/kg CB (CBL), 1×10^9^CFU/kg CB (CBH), 500mg/kg SB, and 300mg/kg BAG. Results showed that the laying performance and egg quality were increased by CBL, CBH, and BAG. Both CB treatments increased the hatchability of fertilized eggs. Maternal supplementation with both levels of CB significantly elevated the growth performance of offspring. Treatment with CBL, CBH, SB, and BAG all improved the oviduct-related variables and reduced the plasmal antioxidant variables. The CBH, CBL, and BAG treatments also improved the intestinal morphology to different degrees. Jejunal contents of IL-6 were decreased by CBH and BAG, while those of IL-4, IL-6, IL-1β, and IgY were decreased by SB. Transcripts of nutrient transporters in jejunal mucosa were also upregulated by CBH, CBL, and SB treatments and expression of Bcl-2-associated X protein was decreased by CBL, CBH, and BAG. In cecal contents, CBL increased the abundance of Firmicutes and Bacillus, while CBH decreased the abundance of Proteobacteria. Also, the co-occurrence networks of intestinal microbes were regulated by CBH and BAG. In conclusion, dietary inclusion of CB and BAG improved the reproductive parameters, egg quality, and intestinal morphology of breeders. CB also influenced the hatching performance of breeders and growth performance of the offspring, while SB improved the oviduct-related variables. These beneficial effects may result from the regulation of cytokines, nutrient transporters, apoptosis, and gut microbiota; high-level CB had more obvious impact. Further study is needed to explore and understand the correlation between the altered gut microbiota induced by butyrate and the performance, egg quality, intestinal health, and also offspring performance.

## Introduction

Butyrate, a short-chain fatty acid (SCFA), is a main end-product of intestinal microbial fermentation of dietary fiber (Xiao et al., 2020). Butyrate is important for the health of animals because it provides nutrition for epithelial cells and helps inhibit pathogens in the gut (Meimandipour et al., 2010).

*Clostridium butyricum* (CB) is a butyric acid-producing Gram-positive anaerobe and has been used as feed additive in poultry industry. Previous studies in chickens suggested that CB promoted growth performance (Zhang et al., 2011; Yang et al., 2012), improved intestinal morphology (Zhang et al., 2011), altered intestinal microbiota (Meimandipour et al., 2010; Yang et al., 2012), and ameliorated inflammation (Zhang et al., 2016; Takahashi et al., 2018). Similarly, sodium butyrate (SB), functioning as butyric acid in the acidic environment of the proximal digestive tract of the birds (Ahsan et al., 2016), also increased the growth performance (Sikandar et al., 2017), meat quality (Deepa et al., 2017; Gomathi et al., 2018), and anti-inflammatory ability of chickens (Jiang et al., 2015). Butyric acid glycerides (BAG), the tributyl ester compound, is an enriched source with fewer undesirable properties. Previous research indicated that BAG effectively improved the growth performance and lipid metabolism of broilers (Taherpour et al., 2009). In the study of Jahanian and Golshadi (2015), the productive performance of laying hens was found to be enhanced by BAG.

Although CB, SB, and BAG promoted the performance and growth of breeder hens, their specific roles and efficacy remained unclear. The present study, therefore, was designed to investigate and compare the effects of CB, SB, and BAG on the reproductive performance, egg quality, intestinal health, and offspring performance of yellow-feathered breeder hens, breeds of major importance in China. In addition, because of apparent altered function of the small intestine, the relative expression of selected nutrient transporter genes, and other relevant transcripts, was measured.

## Materials and Methods

### Breeder Hens and Management

The experimental protocol was approved by the Animal Care Committee of the Institute of Animal Science, Guangdong Academy of Agriculture Science, Guangzhou, China, with the approval number GAASISA-2019-007. A total of 300 Lingnan yellow-feathered breeder hens of 45weeks of age with similar BW (3.02±0.01kg) and laying rate were used.

Breeder hens were randomly allocated into five treatment groups, each with 10 replicates of 6 birds. The trial lasted from 45 to 54weeks of age. During the experimental period, hens were housed singly in laying cages, received 120g of feed per bird per day to prevent over-feeding, and had access to fresh water *ad libitum*. Breeder hens received artificial insemination of 25μl pooled semen per bird every 3days. Twenty males were used and received the basal diet. At the end of the experiment, eggs were collected and incubated as described below.

One hundred hatched chicks, pooled from each treatment, were divided into five replicates and fed a standard diet for 28days in pens (stocking density 0.38m^2^/bird). Daylight was eliminated and replaced with 18-h lighting from incandescent bulbs. The temperature of the room was maintained at 32 to 34°C for the first 3days and then reduced by 2 to 3°C per week to a final temperature of 26°C. Chicks received feed and fresh water *ad libitum*.

### Diets

All breeder hens were fed the same basal diet (Supplementary Table S1) to which 1×10^8^CFU/kg *Clostridium butyricum* (CBL), 1×10^9^CFU/kg *Clostridium butyricum* (CBH), 500mg/kg coated sodium butyrate (SB), and 300mg/kg BAG were added to obtain the treatments. *Clostridium butyricum* (1×10^9^CFU/kg) and coated sodium butyrate (effective content: 40% butyrate) were purchased from Huijia Biological Technology Co., Ltd. (Hangzhou, China). BAG (tributyrin: 45% butyrate) was obtained from Youjiu Biological Technology Co., Ltd. (Shanghai, China). All offspring chicks were fed a common diet (Supplementary Table S2).

### Production Performance and Egg Quality

Egg production, egg weight, and number of qualified eggs were recorded daily. The unqualified eggs included misshapen eggs, dirty eggs, excessively large or small eggs, broken eggs, cracked eggs, and eggs without a shell (but with intact membrane) according to Duan et al. (2015). The laying rate, daily egg mass, average egg weight, and qualified rate of egg were calculated. At 53weeks of age, three eggs per replicate were selected to determine the egg quality, including egg shape index, yolk ratio, yolk color, albumen height, Haugh unit (automatic egg analyzer, EMT-5200 Robotmation Co., Ltd., Tokyo, Japan), eggshell thickness (by micrometer), and eggshell strength (eggshell strength tester, FGV-10XY, Orka Food Technology, Ramat HaSharon, Israel).

At 55weeks of age, 30 eggs from each replicate and 10 replicates from each treatment were selected for hatching. All eggs were incubated in the same incubator (Bengbu Sanyuan Incubation Equipment Co., Ltd., Anhui, China) at 37.2°C to 38.0°C and 60 to 75% relative humidity. Eggs were turned 12 times/day throughout the incubation period and sprayed with water once daily from the 15th day of incubation until they hatched (Xia et al., 2020). The fertility, hatch of fertile eggs and hatchling weight were recorded and calculated.

At the end of the experiment, one hen from each replicate was selected to measure the weight and length of the oviduct, the weight of ovarian stroma, the number and weight of large yellow follicles (LYF), and the number and weight of small yellow follicles (SYF). The oviduct included the funnel part, enlargement, isthmus, and the uterine. The LYF (>8mm) and SYF (3–8mm) were identified by measuring the diameter using the vernier caliper.

The growth performance of the 20 offspring from each replicate was also determined. The average daily gain (ADG) was determined from BW at hatching and day 28. The average daily feed intake was determined from feed consumed by each replicate.

### Biochemical Determinations

At the end of the experiment, hens selected at Section “Production Performance and Egg Quality” were weighed and 6ml heparinized blood was obtained *via* the wing vein. Plasma was obtained by centrifugation at 1,500×*g* for 10min. The birds were then killed by cervical dislocation. Liver, ovary and jejunal mucosa (rinsed and scraped with a glass slide) were collected and snap-frozen in liquid N_2_, homogenized with ice-cold physiologic saline (1:10, v/v), and centrifuged at 2,000×*g* for 10min. The activities of alkaline phosphatase (AKP), total antioxidant capacity (T-AOC), diamine oxidase (DAO), total superoxidase dismutase (T-SOD), and content of uric acid in the plasma and in the supernatants of liver, jejunal mucosa, and ovary were analyzed spectrophotometrically using commercial kits (Nanjing Jiancheng Bioengineering Institute, Nanjing, China).

### Intestinal pH and Morphology

The pH of small intestinal contents was measured with a portable meter equipped with an insertion glass electrode (HI8424, HANA Instrument Science and Technology Co., Ltd., Beijing, China). Duodenum, jejunum, and ileum were collected immediately after slaughter. The intestines were opened with sterile scissors, and pH of the contents was measured by inserting a glass pH electrode probe.

One-centimeter lengths from the medial portions of the jejunum were washed in physiological saline solution and fixed in 10% buffered formalin. Tissue samples were later embedded in paraffin, and a 20-μm sections of each sample were dewaxed, mounted on glass slides, and stained with hematoxylin and eosin. Slides were blindly evaluated microscopically (Eclipse Ti-E, Nikon, Japan). Villus height was measured from the top of the villus to the junction of villus and crypt, and crypt depth was measured as the depth of the invagination between adjacent villi using scanning browsing software (CaseViewer2.2, 3DHISTECH, Hungary) and scanning analysis software (Halo v3.0.311.314, Indica Labs, Albuquerque, NM). For each jejunal sample, the height of five intact villi and the corresponding depths of five crypts were measured and the average value was calculated.

### RT-qPCR

Total RNA extraction from the mucosa of jejunum was performed using TRIzol reagent (RNAiso plus 9109, Takara, Tokyo, Japan) and reverse-transcribed with PrimeScript II 1st Strand cDNA Synthesis Kit (6210A, Takara). Real-time PCR was performed with SYBR PremixExTaq II (Takara) and an ABI 7500 real-time PCR system (Applied Biosystems, Carlsbad, CA), as described by Satoh et al. (2010). The primers used are provided in Supplementary Table S3. Results were normalized to the abundance of β-actin transcripts, and relative quantification was calculated using the 2^−ΔΔCT^ method.

### Immune Variables

Jejunal mucosal samples were homogenized with ice-cold physiologic saline (1:10, v/v) and centrifuged at 2,000×*g* for 10min (Centrifuge 5804R, Eppendorf, Germany). Supernatants were collected, and ELISAs were performed to determine the levels of IL-4, IL-6, IL-1β, TNF-α, IgM, IgA, and IgY using kits (Bio-function Technology Co., Ltd., Beijing, China).

### Microbial Analysis

Bacterial genomic DNA was extracted from cecal contents of breeder hens using the TIANamp Stool DNA Kit (Tiangen, Beijing, China). The V3/V4 region of the 16S ribosomal RNA gene was amplified by using the 341F/805R primer pairs, and the sequencing was performed on an Illumina MiSeq platform (Illumina Inc., San Diego, CA). Raw sequences were filtered and clustered into operational taxonomic unit (OTU) at 97% similarity by QIIME 2 software.^1^ Bacterial OTU representative sequences were assigned to a taxonomic lineage by Ribosomal Database Project classifier based on the Greengenes 13.8 database. Alpha diversity was analyzed by QIIME 2 software. Beta diversity was analyzed and plotted by principal coordinates analysis (PCoA) using the “ggplot2” package of R software. Permutational multivariate analysis of variance (PERMANOVA) was calculated by “vegan” package to determine significant differences in microbial beta diversity among the treatment groups (based on the Bray-Curtis distance matrices). To determine the highly dimensional intestinal microbes and characterize the differences among the treatments, linear discriminant analysis (LDA) effect size (LEfSe) analysis^2^ was used. Co-occurrence networks of microbial communities in different treatments were built based on significant correlations (Spearman’s *R*>0.6 and FDR-adjusted *p*<0.05; Jiao et al., 2016) and were visualized by Gephi software (version 0.9.2). The topological properties of networks were calculated to describe the complex patterns of the interrelationships. All the DNA datasets have been submitted to the NCBI Sequence Read Archive database under the BioProject ID: PRJNA695347.

### Statistical Analysis

Replicate served as the experimental unit. The effects of dietary CB, SB, and BAG were analyzed by one-way ANOVA (SPSS Inc., Chicago, IL). Means were separated by Duncan’s multiple range test. Tabulated results are shown as means with SEM derived from the ANOVA error mean square.

## Results

### Laying Performance of Yellow-Feathered Breeder Hens

As shown in Table 1, supplementation with high or low-level CB and BAG increased the laying rate (*p*<0.05). CBH and BAG also increased the daily egg mass (*p*<0.05). The feed:egg ratio was decreased by the high and low level of CB and by BAG (*p*<0.05).

**Table 1:** Laying performance of yellow-feathered breeder hens.

	CL	CBH	CBL	SB	BAG	SEM	Value of *p*
Laying rate, %	50.82^b^	57.10^a^	56.24^a^	54.65^ab^	58.61^a^	0.90	0.021
Daily egg mass, g	30.60^b^	34.75^a^	33.81^ab^	32.89^ab^	34.68^a^	0.53	0.041
Average egg weight, g	60.90	61.70	60.73	60.71	60.09	0.58	0.841
Qualified rate of egg, %	94.68	97.31	96.27	93.97	96.57	0.45	0.111
Feed/egg	3.50^a^	3.05^b^	3.15^b^	3.24^ab^	3.06^b^	0.05	0.039

ab *Means within a row lacking a common superscript differ significantly (p<0.05)*.

*CL, control; CBL, low-level Clostridium butyricum; CBH, high-level Clostridium butyricum; SB, sodium butyrate; BAG, butyric acid glycerides; and SEM, standard error*.

### Hatching Performance and Egg Quality of Yellow-Feathered Breeder Hens

According to Table 2, CBL, CBH, SB, and BAG had no significant effects on the fertility, hatch of fertile eggs, and hatchling weight, compared to the controls (CL). Birds in the CBL treatment had lower hatchability of fertilized eggs (*p*<0.05) than those receiving CBH. Significantly, increased (*p*<0.05) albumen height and eggshell thickness were noted for the CBH treatment and CBL increased the albumen height (*p*<0.05; Table 3). The BAG treatment increased (*p*<0.05) the yolk color and Haugh unit.

**Table 2:** Hatching performance of yellow-feathered breeder hens.

	CL	CBH	CBL	SB	BAG	SEM	Value of *p*
Fertility, %	85.33	80.00	81.33	82.00	86.67	1.76	0.823
Hatch of feritile eggs, %	95.59^ab^	97.50^a^	89.53^b^	90.89^ab^	96.05^ab^	1.08	0.029
Chick weight, g	44.14	44.79	43.67	42.91	42.65	0.31	0.061

ab *Means within a row lacking a common superscript differ significantly (p<0.05)*.

*CL, control; CBL, low-level Clostridium butyricum; CBH, high-level Clostridium butyricum; SB, sodium butyrate; BAG, butyric acid glycerides; and SEM, standard error*.

**Table 3:** Egg quality of yellow-feathered breeder hens.

	CL	CBH	CBL	SB	BAG	SEM	Value of *p*
Egg shape index	1.31	1.27	1.32	1.28	1.32	0.01	0.066
Yolk ratio, %	32.68	31.20	31.11	31.99	32.13	0.23	0.206
Yolk color	5.41^b^	5.27^b^	5.35^b^	5.29^b^	6.11^a^	0.10	0.033
Albumen height, mm	6.65^b^	7.33^a^	7.54^a^	6.75^b^	6.80^b^	0.11	0.043
Haugh unit	70.97^b^	69.44^b^	70.10^b^	70.45^b^	76.50^a^	0.82	0.044
Eggshell thickness, mm	0.30^b^	0.34^a^	0.31^b^	0.32^ab^	0.32^ab^	0.00	0.001
Eggshell strength, kg/cm^2^	3.20	3.32	3.29	3.34	3.57	0.06	0.497

ab *Means within a row lacking a common superscript differ significantly (p<0.05)*.

*CL, control; CBL, low-level Clostridium butyricum; CBH, high-level Clostridium butyricum; SB, sodium butyrate; BAG, butyric acid glycerides; and SEM, standard error*.

### Oviduct-Related Variables of Yellow-Feathered Breeder Hens

Compared with the controls, CBH treatment increased (*p*<0.05) the weight and length of the oviduct, and number and weight of LYF. Number and weight of LYF also increased (*p*<0.05) by CBL, SB increased weight of the oviduct, and number and weight of LYF, while BAG treatment increased (*p*<0.05) the number of LYF (Table 4).

**Table 4:** Oviduct-related variables of yellow-feathered breeder hens.

	CL	CBH	CBL	SB	BAG	SEM	Value of *p*
Weight of oviduct/BW, %	0.96^c^	1.39^ab^	1.11^bc^	1.26^ab^	1.12^abc^	0.05	0.022
Length of oviduct, cm	34.67^b^	39.10^a^	37.67^ab^	37.70^ab^	39.00^ab^	0.73	0.048
Weight of ovarian stroma/BW, %	0.21	0.26	0.26	0.24	0.23	0.01	0.108
LYF number	3.00^b^	4.10^a^	4.30^a^	4.10^a^	4.30^a^	0.14	0.034
LYF weight/BW, %	0.82^b^	1.28^a^	1.52^a^	1.50^a^	1.22^ab^	0.07	0.022
SYF number	12.11	8.80	11.50	9.10	10.90	0.60	0.230
SYF weight/BW, %	0.07	0.06	0.09	0.05	0.06	0.01	0.165

abc *Means within a row lacking a common superscript differ significantly (p<0.05)*.

*BW, body weight; LYF, large yellow follicles, follicles with mean diameter >8mm; SYF, small yellow follicles, follicles with mean diameter of 3 to 8mm. CL, control; CBL, low-level Clostridium butyricum; CBH, high-level Clostridium butyricum; SB, sodium butyrate; BAG, butyric acid glycerides; and SEM, standard error*.

### Biochemical Variables of Yellow-Feathered Breeder Hens

Treatments with CBH, CBL, and SB all decreased (*p*<0.05) T-AOC activity and uric acid concentration, CBH also lowered plasmal T-SOD activity (*p*<0.05), and BAG treatment significantly decreased T-AOC and T-SOD activity (Table 5). There were no effects of treatment on the activities of T-AOC and T-SOD in jejunal mucosa, liver, or ovary (Table 6).

**Table 5:** Biochemical variables of yellow-feathered breeder hens.

	CL	CBH	CBL	SB	BAG	SEM	Value of *p*
Plasma							
AKP, U/ml	6.33	6.53	6.34	5.67	5.76	0.20	0.741
T-AOC, U/ml	6.94^a^	5.10^b^	5.22^b^	4.94^b^	5.82^b^	0.17	0.003
DAO, U/ml	16.41	19.99	19.52	17.64	16.33	0.91	0.707
T-SOD, U/ml	29.36^a^	22.57^b^	24.66^ab^	24.19^ab^	21.74^b^	0.79	0.047
Uric acid, mmol/ml	357.84^a^	303.48^b^	302.07^b^	297.24^b^	344.62^ab^	7.80	0.036
Jejunum							
T-SOD, U/mg pro	2086.05^ab^	1982.63^ab^	2560.30^a^	1889.25^b^	2379.53^ab^	93.97	0.028
T-AOC, U/mg pro	21.09	23.55	20.06	26.93	16.02	1.51	0.430
Liver							
T-SOD, U/mg pro	1198.71^b^	1190.07^b^	1333.98^a^	1184.43^b^	1101.78^b^	36.12	0.018
T-AOC, U/mg pro	3.05	3.79	4.58	3.95	4.03	0.16	0.320
Ovary							
T-SOD, U/mg pro	5086.97	5663.54	4794.13	6008.14	5365.03	248.80	0.633
T-AOC, U/mg pro	1.60	1.48	2.46	2.65	1.88	0.42	0.231
MDA, nmol/mg pro	5.34	4.55	2.73	3.31	3.53	0.43	0.509

ab *Means within a row lacking a common superscript differ significantly (p<0.05)*.

*AKP, alkaline phosphatase; T-AOC, total antioxidant capacity; DAO, diamine oxidase; T-SOD, total superoxidase; MDA, malondialdehyde; CL, control; CBL, low-level Clostridium butyricum; CBH, high-level Clostridium butyricum; SB, sodium butyrate; BAG, butyric acid glycerides; and SEM, standard error*.

**Table 6:** Intestinal pH and morphology of yellow-feathered breeder hens.

	CL	CBH	CBL	SB	BAG	SEM	Value of *p*
pH							
Duodenum	6.32	6.22	6.33	6.38	6.35	0.03	0.601
Jejunum	6.34	6.31	6.21	6.29	6.31	0.02	0.522
Ileum	6.66	6.72	6.61	6.65	6.55	0.03	0.249
Morphology of jejunum							
Villus height, mm	0.91^b^	1.24^a^	1.08^a^	0.92^b^	1.11^a^	0.02	0.026
Crypt depth, mm	0.14^b^	0.25^a^	0.17^b^	0.18^b^	0.21^a^	0.01	0.043
Villous crypt ratio	5.35^b^	7.56^a^	6.67^a^	5.08^b^	5.49^b^	0.18	0.016

^ab^*Means within a row lacking a common superscript differ significantly (p<0.05)*.

*CL, control; CBL, low-level Clostridium butyricum; CBH, high-level Clostridium butyricum; SB, sodium butyrate; BAG, butyric acid glycerides; and SEM, standard error*.

### Intestinal Morphology of Yellow-Feathered Breeder Hens

The pH of duodenal, jejunal, and ileal digesta was not significantly influenced by treatment (*p*>0.05). Jejunal morphology was improved by CBH, CBL, and BAG. As shown in Table 6, CBH increased (*p*<0.05) the villus height, crypt depth, and the villus:crypt ratio, CBL increased (*p*<0.05) the villus height and villus:crypt ratio, and BAG increased (*p*<0.05) the villus height and crypt depth of jejunal mucosa.

### Immune Factors of Yellow-Feathered Breeder Hens

The contents of cytokines and immunoglobulins in the jejunal mucosa were determined (Table 7). The only effect on IL-4 was the reduction (*p*<0.05) caused by SB treatment. Contents of IL-6 were reduced (*p*<0.05) by CBH, SB, and BAG. The SB treatment also reduced IL-1β and IgY, the latter quite strikingly, and SB also resulted in lowest contents of IgA.

**Table 7:** Jejunal immune factors of yellow-feathered breeder hens.

	CL	CBH	CBL	SB	BAG	SEM	Value of *p*
IL-4 pg/mg pro	9.18^a^	7.53^ab^	8.91^a^	6.53^b^	7.30^ab^	0.32	0.068
IL-6 μg/mg pro	2.04^a^	1.39^b^	1.69^ab^	1.36^b^	1.54^b^	0.16	0.011
IL-1β ng/mg pro	3.86^a^	3.80^a^	3.99^a^	3.31^b^	3.94^a^	0.31	0.020
TNF-α ng/mg pro	9.70	9.28	9.53	7.82	9.83	0.86	0.414
IgM μg/mg pro	10.16	10.63	12.07	9.03	10.49	1.27	0.297
IgA μg/mg pro	12.56^ab^	12.16^ab^	14.55^a^	10.87^b^	13.45^ab^	1.09	0.043
IgY μg/mg pro	150.22^a^	134.93^ab^	159.60^a^	117.26^b^	141.16^ab^	4.68	0.018

ab *Means within a row lacking a common superscript differ significantly (p<0.05)*.

*IL-4, interleukin 4; IL-6, interleukin 6; IL-1β, interleukin 1β; TNF-α, tumor necrosis factor α; IgM, immunoglobulin M; IgA, immunoglobulin A; IgY, immunoglobulin Y; CL, control; CBL, low-level Clostridium butyricum; CBH, high-level Clostridium butyricum; SB, sodium butyrate; BAG, butyric acid glycerides; and SEM, standard error*.

### Jejunal Gene Expression of Yellow-Feathered Breeder Hens

The relative transcript abundance of Na(+)/H(+) exchanger isoform 2 (*NHE2*) was increased (*p*<0.05) by CBL and SB, that of peptide transporter 1 (*PEPT1*) by CBL and SB, glucose transporter-2 (*GLUT2*) by both CBH and CBL, and apical nutrient transporter SLC6A19 (*B^0^AT*) was increased only by CBH. The apoptosis-related gene Bcl-2-associated X protein (*BAX*) was downregulated (*p*<0.05) by CBH, CBL, and BAG; the expression of *TNF-α* was not significantly affected by treatment (Table 8).

**Table 8:** Jejunal gene expressions of yellow-feathered breeder hens.

	CL	CBH	CBL	SB	BAG	SEM	Value of *p*
*NHE2*	0.91^c^	1.53^abc^	2.35^a^	1.95^ab^	1.39^bc^	0.28	0.018
*PEPT1*	1.03^b^	1.79^a^	0.70^b^	2.14^a^	1.47^ab^	0.33	<0.001
*GLUT2*	0.84^b^	1.72^a^	1.72^a^	0.48^b^	0.45^b^	0.35	<0.001
*B^0^AT*	0.92^b^	2.39^a^	1.22^b^	1.73^ab^	1.14^b^	0.37	0.018
*BAX*	1.30^a^	0.66^b^	0.58^b^	0.87^ab^	0.64^b^	0.18	0.036
*TNF-α*	0.99	1.11	1.32	0.97	0.87	0.19	0.053

abc *Means within a row lacking a common superscript differ significantly (p<0.05)*.

*NHE2, Na(+)/H(+) exchanger isoform 2; PEPT1, peptide transporter 1; GLUT 2, glucose transporter-2; B^0^AT, apical nutrient transporter SLC6A19; BAX, Bcl-2 associated X protein; TNF-α, tumor necrosis factor α; CL, control; CBL, low-level Clostridium butyricum; CBH, high-level Clostridium butyricum; SB, sodium butyrate; BAG, butyric acid glycerides; and SEM, standard error*.

### Gut Microbiota Structure of Yellow-Feathered Breeder Hens

**Figure 1:**
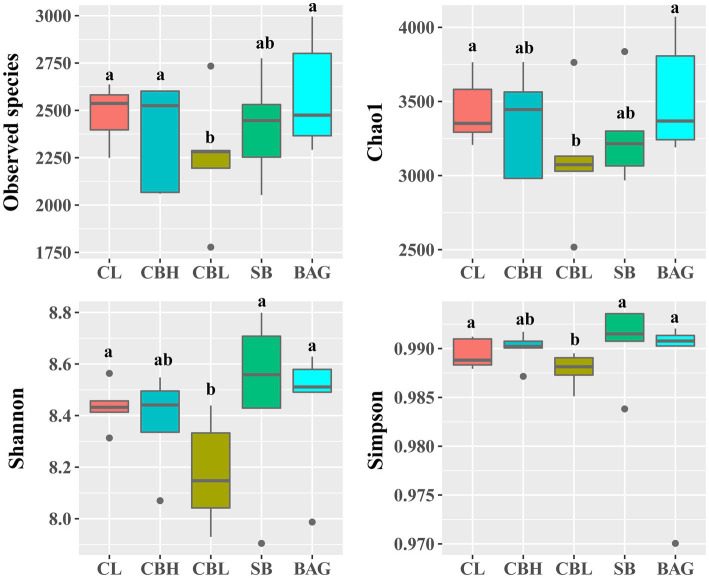
Alpha diversity analysis of intestinal microbiota in different treatments.

**Figure 2:**
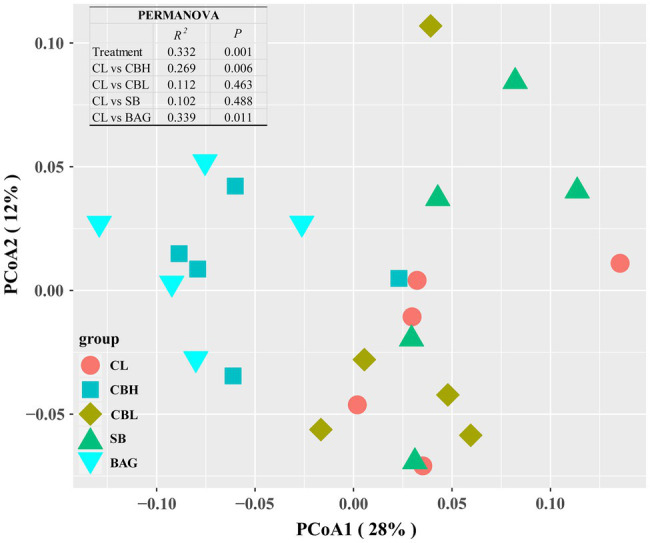
Principal coordinates analysis (PCoA) of microbial communities among treatment groups based on Bray-Curtis distance.

**Figure 3:**
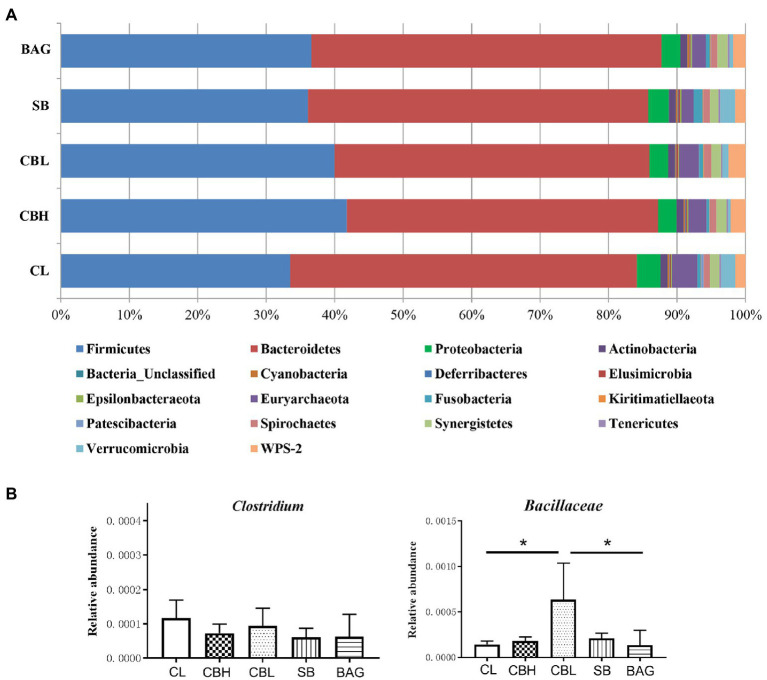
Relative abundance of gut microbiota. **(A)** Relative abundance of gut microbiota at phylum level. **(B)** Relative abundance of *Clostridium* and *Bacillaceae*.

The results showed that the CBL treatment decreased the variables of the observed species, Chao1, Shannon and Simpson (*p*<0.05) compared to the controls (Figure 1). PCoA of intestinal microbiota based on Bray-Curtis distance revealed distinct clusterings of the gut microbiota composition between CL and CBH, and between CL and BAG (Figure 2). The differences in the intestinal bacterial compositions between the treatments were also analyzed. The results showed that at the phylum level, CBH and CBL treatments obviously increased the abundance of Firmicutes. Additionally, CBH also decreased the abundance of Proteobacteria. At the family level, CBL increased (*p*<0.05) the abundance of Bacillaceae over those of the CL and BAG treatments. At the genus level, no significant treatment differences were noticed in the abundance of *Clostridium* (Figure 3).

**Figure 4:**
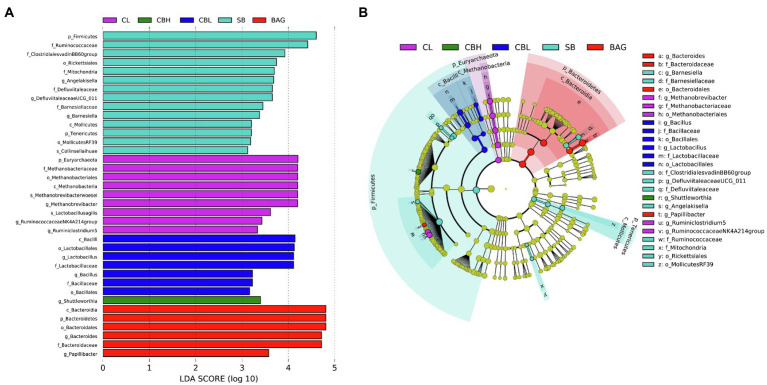
Linear discriminant analysis (LDA) effect size (LEfSe) analysis (LDA>3.0, *p*<0.05) of intestinal microbes. The prefixes “p_,” “c_,” “o_,” “f_,” “g_,” and “s_” represent the annotated level of phylum, class, order, family, genus, and species. **(A)** LDA and **(B)** LEfSe.

In order to further identify microbial taxa that account for the greatest differences between genders, we performed LDA coupled with effect size measurements (LEfSe). In the total cohort, families with higher abundance in the CL controls included Methanobrevibacter and Ruminiclostridium5. The genus *Shuttleworthia* was higher in the CBH treatment. Bacillaceae, Bacillales, *Bacillus* and Lactobacillaceae, Lactobacillales, and *Lactobacillus* were more abundant in the CBL treatment. The SB treatment induced higher abundances of Barnesiellaceae, ClostridialesvadinBB60, Defluviitaleaceae, *Angelakisella*, Ruminococcaceae, and Mitochondria. Moreover, more abundant Bacteroidales, Bacteroidaceae, Bacteridales, and *Bacteroides* were observed in BAG treatment (Figure 4).

**Figure 5:**
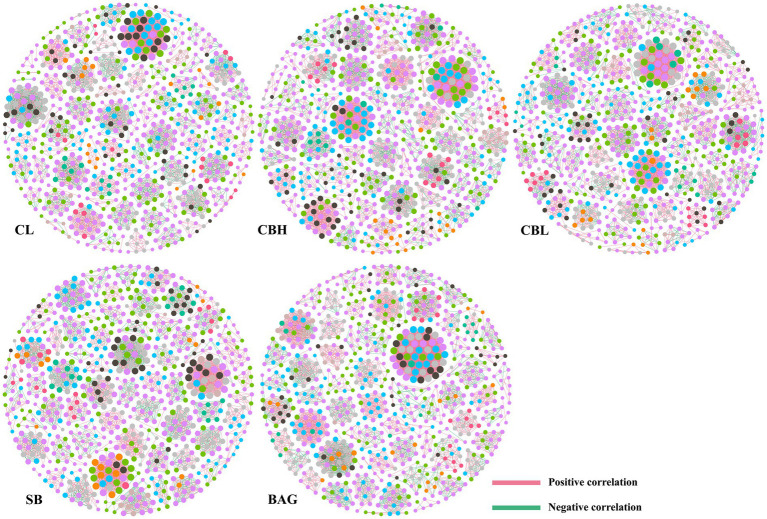
Co-occurrence networks of microbial communities at OTU level. A connection stands for a very strong (Spearman’s *R*>0.6) and significant (FDR-adjusted *p*<0.05) correlation. The size of each node is proportional to the relative abundance; the thickness of each connection between two nodes (edge) is proportional to the value of Spearman’s correlation coefficients. Pink lines represent significant positive correlations, and green lines denote negative correlations.

To investigate the co-occurrence patterns of intestinal microbes in the groups, three networks were constructed based on the OTU level (Figure 5 and Table 9). Co-occurrence network analysis showed that the microbial networks were roughly at the same edges and nodes among the different treatments. The values of average degree (AD) in breeder hens treated with CBH and BAG were higher than those in the controls; the graph density (GD) and the modularity (MD) values were not altered obviously among groups. Additionally, the negative correlations of the network in the CBH and BAG treatments were more than that of the controls while the positive correlations of the network were less than that of the controls.

**Table 9:** Topological properties of co-occurrence network.

	CL	CBH	CBL	SB	BAG
Nodes	763	756	768	766	786
Edges	4,221	4,552	4,259	4,160	4,919
AD	11.064	12.042	11.091	10.862	12.517
GD	0.015	0.016	0.014	0.014	0.016
MD	0.955	0.954	0.957	0.968	0.93
Positive correlation	69.30%	75.00%	69.00%	69.00%	78.00%
Negative correlation	30.70%	24.00%	30.00%	30.00%	21.00%

*AD, average degree; GD, graph density; and MD, modularity*.

### Growth Performance of Offspring Broilers

Compared to the control breeders, CBH breeders produced offspring with increases (*p*<0.05) in the final BW and ADG, and with decreased (*p*<0.05) feed:gain. The feed:gain of offspring from the CBL treatment was also decreased (*p*<0.05; Table 10).

**Table 10:** Offspring performance of yellow-feathered breeder hens.

	CL	CBH	CBL	SB	BAG	SEM	Value of *p*
Final body weight, g	700.08^b^	745.80^a^	722.00^ab^	702.50^b^	713.00^ab^	6.09	0.037
Average daily gain, g	44.98^b^	45.65^a^	44.37^ab^	43.85^b^	44.78^ab^	0.22	0.037
Total daily feed intake, g	1259.48	1278.10	1242.30	1227.80	1253.90	15.37	0.155
Feed/gain	1.81^a^	1.71^b^	1.72^b^	1.75^ab^	1.76^ab^	0.01	0.019

ab *Mean values within a row with no common superscript differ significantly (p<0.05)*.

*CL, control; CBL, low-level Clostridium butyricum; CBH, high-level Clostridium butyricum; SB, sodium butyrate; BAG, butyric acid glycerides; and SEM, standard error*.

## Discussion

Evidence exists for butyric acid exerting beneficial effects on the laying and hatching performances of hens (Jahanian and Golshadi, 2015; Zhan et al., 2019). In accordance with these findings, in the current study, CB and BAG were also found to increase the laying rate as well as daily egg weight, while decreasing the feed:egg ratio of yellow-feathered breeder hens. Additionally, because butyrate promoted the absorption and utilization of minerals including calcium (Soltan, 2008; Casselbrant et al., 2020), egg quality might be improved by dietary addition of butyric acid. Eggshell strength (Zhan et al., 2019) and yolk color (Wang et al., 2020) were increased by dietary CB. In the present study, parameters of egg quality, such as albumen height and eggshell thickness, were also improved by CB, and yolk color and Haugh unit were elevated by BAG. It was noteworthy that the offspring broilers from breeders fed the CBH diet had a much better growth performance than that in other treatments.

The hen oviduct was of special interest to commercial egg producers because disrupted activity or pathological changes directly affected egg quality and ultimately decreased economic profitability (Chousalkar and Roberts, 2008). In the present study, the oviduct-related variables, such as weight of the oviduct, and number and weight of LYF, were improved by the treatment of CB, SB, or BAG. Little was known about the effects of butyrate on oviduct-related variables to serve for comparison with the present results. However, according to Ghosh and Cox (1977), SB induced the secretion of follicle-stimulating hormone (FSH), which stimulated the growth and development of follicles. Thus, butyrate might promote the reproductive ability and egg quality through regulating oviduct development by inducing FSH secretion.

It is reported that oxidative stress decreased the hatchability and increased the mortality post-hatch (Nadia et al., 2008). Thus, the elevated hatchability and reproductive performance in the present study prompted us to investigate the oxidative status of breeder hens among the different treatments. It was reported that dietary provision of CB promoted the serum, hepatic, and intestinal antioxidant status of hosts (Liao et al., 2015; Duan et al., 2017; Zhan et al., 2019) and SB also improved antioxidant capacity *in vitro* and *in vivo* (Xing et al., 2016; Honma et al., 2020). It was found here, however, that CB, SB, and BAG had minimal effect on the activities of T-SOD and T-AOC in jejunal mucosa, liver, and ovary; SB did reduce jejunal T-SOD activity. Plasmal activity of T-AOC was decreased by CB, SB, and BAG treatments, and activity of T-SOD was reduced by BAG and CBH. As the performance of yellow-feathered breeder hens in the current study was significantly improved, the decreased antioxidant variables might be due to the decreased expression of reactive oxygen species-generating proteins, rather than the impairment of the antioxidant system. The plasmal content of uric acid was also reduced by treatment with CBH, CBL, and SB. Uric acid was the major final product of nitrogen metabolism in birds and was an endogenous antioxidant (Sauer et al., 2007). In the study of Al-Yasiry et al. (2017), dietary supplementation with *Boswellia serrata* resin, a putative anti-inflammatory agent, improved the performance of broiler chickens and decreased uric acid. Decreased plasmal content of uric acid might suggest greater efficiency of use of absorbed amino acids or reduced turnover of endogenous proteins in broiler chickens (Scanes, 2015; Al-Yasiry et al., 2017). Moreover, oxidative stress played a pivotal role in apoptosis and the elevation of antioxidant status could block or delay apoptosis (Kannan and Jain, 2000). It was found here that the expression of the pro-apoptosis gene *BAX* was downregulated by CBH, CBL, and BAG, but not by SB, suggesting that CB and BAG likely attenuated apoptosis.

Relationships were found between reproductive performance and immune function. For example, TLR4 polymorphisms offered a meaningful tool to judge the reproductive potential (Shimizu et al., 2017). The immunostimulatory property of butyrate in chickens was known (Sunkara et al., 2011). Zhou et al. (2014) discovered that SB suppressed the expression of *IL-1β*, *IL-6*, *IFN-γ*, and *IL-10* in chicken macrophages stimulated by *S. typhimurium* LPS. The present study also showed that SB reduced jejunal contents of IL-4, IL-6, and IL-1β. The CBH treatment decreased jejunal content of IL-6, supporting recent work that CB administration reduced IL-6 level in the intestine of burned mice (Zhang et al., 2020). Immunoglobulins are key immune mediators. Levels of IgM and IgA in jejunal mucosa were not altered by CB, SB, or BAG, but IgY level was reduced by SB. Previous studies have suggested that SB increased IgY to improve immune function. For instance, Makled et al. (2019) found that meat-type chickens receiving a SB diet increased IgY, and Gong et al. (2020) showed that β-carotene, curcumin, allicin, and SB supplementation increased serum IgY in breeder hens. The decreased jejunal IgY of SB-treated breeder hens in the present study may indicate weakened humoral immunity. Altogether, the above findings indicated that CBH, SB, and BAG affected jejunal cytokines and SB might have an adverse effect on humoral immunity of breeder hens.

Interactions between nutrition and immunity were diverse and had profound implications on animal productivity. A supply of nutrients at the appropriate times and amounts was important for the immune system (Humphrey and Klasing, 2004). Nutrient transporters mediated substrate-specific uptake across the plasma membrane. Reports showed that SCFA enhanced the structural and functional adaptation of the mammalian intestine by increasing transcript abundance of *GLUT2* (Tappenden et al., 1997; Tappenden and McBurney, 1998; Mangian and Tappenden, 2009), mediating one of the major pathways of intestinal sugar absorption (George and Edith, 2005). In the present study, both CBH and CBL increased *GLUT2* transcripts in the avian jejunal mucosa. Electroneutral Na absorption in the intestine used NHE isoforms NHE2 and NHE3. Butyrate was reported to stimulate electroneutral Na absorption through NHE2 (Subramanya et al., 2007). In the current study, CBL and SB also upregulated the jejunal expression of *NHE2*. Protein was hydrolyzed to small peptides and amino acids in the small intestine, and dipeptides and tripeptides were transported across enterocytes *via* Pept1 or were catabolized to amino acids by aminopeptidase N (Wang et al., 2020). Dalmasso et al. (2008) reported that butyrate enhanced the expression and activity of Pept 1 in mice. Liu et al. (2017) found that SB supplementation induced *Pept1* expression in juvenile grass carp. Consistent with these previous studies, the present results showed that CBH and SB upregulated the expression of *Pept1*. B^0^AT is a Na^+^-dependent neutral amino acid transporter (Wang et al., 2020). The present authors were unaware of any exploration of the effect of butyrate on the expression of *B^0^AT*, and it was found here that *B^0^AT* transcripts were induced by CBL. Hence, the above findings demonstrated that CB and SB were likely able to increase capacity for the absorption of nutrients.

Gut microbiota affected the parameters of fitness, such as survival, phenotypic plasticity, and reproductive performance of animals (Zilber-Rosenberg and Rosenberg, 2008). Dietary butyrate functioned as a bactericidal agent because it can lower the pH in the crop and gizzard and proximal intestine, to control harmful bacteria, such as *Salmonella* spps., *Escherichia coli*, and *Campylobacter jejuni* (Van Deun et al., 2008). Hens provided with CB had reduced cecal *E. coli* and increased *Bifidobacterium* (Zhan et al., 2019). Czerwiński et al. (2012) found that dietary SB increased total bacterial numbers and *Lactobacillus* counts in ileal and cecal contents of chickens. Supplementation of laying hens reduced total bacteria and *ileal E. coli* numbers (Jahanian and Golshadi, 2015). In the present study, the cecal microbiota structure of the yellow-feathered breeder hens was examined. Compared with the controls, breeder hens given CBH and CBL had more Firmicutes at the phylum level. As CB belonged to Bacillaceae family and *Clostridium* genus, the relative abundance of Bacillaceae and *Clostridium* was further determined. The present results suggested that CBL increased the presence of Bacillaceae, though abundance of *Clostridium* was unaffected by CB, SB, and BAG treatments. Increased Firmicutes and Bacillaceae in CB treatments might simply stem from the dietary addition of CB. *Firmicutes* bacteria provide a good index of the state of the intestine. For example, Bacillaceae, *Lactobacillus*, and *Lactococcus* could suppress the production of inflammatory cytokines and pathogen-induced disruption of intestine function (Li et al., 2009; Zheng et al., 2017; Dowdell et al., 2020). Dietary CB supplementation of young pigs promoted growth performance and increased the *Bacillus* level (Chen et al., 2018). It is likely, therefore, that elevated abundance of Firmicutes and Bacillaceae induced by CB might promote the growth and health of yellow-feathered breeder hens. Additionally, in agreement with the study of Duan et al. (2018), the abundance of Proteobacteria in the shrimp *Litopenaeus vannamei* was reduced by diets containing 2.5 and 5.0×10^9^CFU/kg, and CBH here, in breeder hens, also decreased the abundance of *Proteobacteria*. Thus, the altered population structure of the gut microbiota caused by dietary CB might contribute to the improved performance of yellow-feathered breeder hens.

LDA and LEfSe analysis showed increased *Shuttleworthia*, *Lactobacillus*, Barnesiellaceae, and *Bacteroides* in CBH, CBL, SB, and BAG treatments, respectively. *Shuttleworthia* had a relationship with carbohydrate and lipid metabolic pathways and thus contributed to weight gain and growth performance in broiler chickens (Lee et al., 2017). *Lactobacillus* generally improved the gastrointestinal tract environment and protected the gut from pathogens (Dicks and Botes, 2010). Moreover, although the roles of Barnesiellaceae were not well known, research suggested that the Behçet’s disease and the use of antibiotic led to decreased abundance of Barnesiellaceae (van der Houwen et al., 2020). Additionally, meta-analysis revealed that lower level of *Bacteroides* was associated with intestinal inflammation (Zhou and Zhi, 2016). Finally, co-occurrence patterns of intestinal microbes were employed to investigate the microbial interactions. In the present study, the positive correlation of the microbial networks in the CBH and BAG treatments was more than those in the controls, while the negative correlation of the microbial networks in the CBH and BAG groups was less than those in controls, which could be interpreted as a reduction in competitive relationships within intestinal microbes (Fan et al., 2018).

## Conclusion

In conclusion, the supplementation with CB and BAG in the diets of breeder hens increased the reproductive performance and improved the egg quality and intestinal morphology. CB treatment also promoted the hatching performance of breeder hens and growth performance of offspring. These effects might result from the regulation of cytokines, nutrient transporters, apoptosis, and profiles of gut microbiota. The beneficial effects described were more obvious with CBH treatment than the CBL treatment, as CBH induced the higher hatchability, eggshell thickness, jejunal crypt depth, and *B^0^AT* expression. Although SB had no beneficial effect on reproductive performance, egg quality, or intestinal morphology, it did impact the oviduct-related variables, nutrient transporters, and immune factors, but the decreased IgY might indicate an adverse influence of SB in breeder hens.

## Data Availability Statement

The datasets presented in this study can be found in online repositories. The names of the repository/repositories and accession number(s) can be found in the article/Supplementary Material.

## Ethics Statement

The animal study was reviewed and approved by the Animal Care Committee of the Institute of Animal Science, Guangdong Academy of Agriculture Science, Guangzhou, China, with the approval number of GAASISA-2019-007.

## Author Contributions

YiW and YaW designed the experiments. QF and XL performed animal husbandry. ZG and SJ analyzed 16S rRNA data. YiW did the animal experiments. YaW and SJ wrote the final article. All authors contributed to the article and approved the submitted version.

## Funding

This work was financially supported by the China Agriculture Research System of MOF and MARA (CARS-41), National Natural Science Foundation of China (3180131530), Foundation from Guangdong Province (2021A1515012412 and 2021A1515010830), the Science and Technology Program of Guangdong Academy of Agricultural Sciences (202106TD, R2019PY-QF008 and R2018QD-076), China and the Key Realm R&D Program of Guangdong Province (2020B0202090004).

## Conflict of Interest

The authors declare that the research was conducted in the absence of any commercial or financial relationships that could be construed as a potential conflict of interest.

## Publisher’s Note

All claims expressed in this article are solely those of the authors and do not necessarily represent those of their affiliated organizations, or those of the publisher, the editors and the reviewers. Any product that may be evaluated in this article, or claim that may be made by its manufacturer, is not guaranteed or endorsed by the publisher.

## Footnotes

^1^ https://qiime2.org

^2^ https://huttenhower.sph.harvard.edu/galaxy/

## References

[ref1] Ahsan U. Cengiz O. Raza I. Kuter E. Chacher M. F. A. Iqbal Z.. (2016). Sodium butyrate in chicken nutrition: the dynamics of performance, gut microbiota, gut morphology, and immunity. World Poult. Sci. J. 72, 265–275. doi: 10.1017/S0043933916000210

[ref2] Al-Yasiry A. R. M. Kiczorowska B. Samolinska W. Kowalczuk-Vasilevand E. Kowalczyk-Pecka D. (2017). The effect of Boswellia serrata resin diet supplementation on production, hematological, biochemical and immunological parameters in broiler chickens. Animal 11, 1890–1898. doi: 10.1017/S1751731117000817, PMID: 28436338

[ref3] Casselbrant A. Fndriks L. Wallenius V. (2020). Glycocholic acid and butyrate synergistically increase vitamin D-induced calcium uptake in Caco-2 intestinal epithelial cell monolayers. Bone Rep. 13:100294. doi: 10.1016/j.bonr.2020.100294, PMID: 32715032 PMC7371747

[ref4] Chen L. Li S. Zhang J. Li W. Jiang X. Zhao X.. (2018). Effects of dietary *Clostridium butyricum* supplementation on growth performance, intestinal development, and immune response of weaned piglets challenged with lipopolysaccharide. J. Anim. Sci. Biotechnol. 9:62. doi: 10.1186/s40104-018-0275-8, PMID: 30159141 PMC6106813

[ref5] Chousalkar K. K. Roberts J. R. (2008). Ultrastructural changes in the oviduct of the laying hen during the laying cycle. Cell Tissue Res. 332, 349–358. doi: 10.1007/s00441-007-0567-3, PMID: 18236079

[ref6] Czerwiński J. Højberg O. Smulikowska S. Engberg R. M. Mieczkowska A. (2012). Effects of sodium butyrate and salinomycin upon intestinal microbiota, mucosal morphology and performance of broiler chickens. Arch. Anim. Nutr. 66, 102–116. doi: 10.1080/1745039X.2012.663668, PMID: 22641923

[ref7] Dalmasso G. Nguyen H. T. T. Yan Y. Charrier-Hisamuddin L. Sitaraman S. V. Merlin D. (2008). Butyrate transcriptionally enhances peptide transporter PepT1 expression and activity. PLoS One 3:e2476. doi: 10.1371/journal.pone.0002476, PMID: 18575574 PMC2423477

[ref8] Deepa K. Purushothaman M. R. Vasanthakumar P. Sivakumar K. (2017). Serum biochemical parameters and meat quality influenced due to supplementation of sodium butyrate in broiler chicken. Int. J. Livest. Res. 7, 108–116. doi: 10.5455/ijlr.20170610051212

[ref9] Dicks L. M. T. Botes M. (2010). Probiotic lactic acid bacteria in the gastro-intestinal tract: health benefits, safety and mode of action. Benef. Microbes 1, 11–29. doi: 10.3920/BM2009.0012, PMID: 21831747

[ref10] Dowdell P. Chankhamhaengdecha S. Panbangred W. Janvilisri T. Aroonnual A. (2020). Probiotic activity of *Enterococcus faeciu*m and *Lactococcus lacti*s isolated from Thai fermented sausages and their protective effect against *Clostridium difficile*. Probiotics Antimicrob. Proteins 12, 641–648. doi: 10.1007/s12602-019-09536-7, PMID: 30888623 PMC7306037

[ref11] Duan X. Li F. Mou S. Feng J. Liu P. Xu L. (2015). Effects of dietary L-arginine on laying performance and anti-oxidant capacity of broiler breeder hens, eggs, and offspring during the late laying period. Poult. Sci. 94, 2938–2943. doi: 10.3382/ps/pev283, PMID: 26467009

[ref12] Duan Y. Wang Y. Dong H. Ding X. Liu Q. Li H.. (2018). Changes in the intestine microbial, digestive, and immune-related genes of *Litopenaeus vannamei* in response to dietary probiotic *Clostridium butyricum* supplementation. Front. Microbiol. 9:2191. doi: 10.3389/fmicb.2018.02191, PMID: 30283419 PMC6156435

[ref13] Duan Y. Zhang Y. Dong H. Wang Y. Zhang J. (2017). Effect of the dietary probiotic *Clostridium butyricum* on growth, intestine antioxidant capacity and resistance to high temperature stress in kuruma shrimp *Marsupenaeus japonicus*. J. Therm. Biol. 66, 93–100. doi: 10.1016/j.jtherbio.2017.04.004, PMID: 28477915

[ref14] Fan K. K. Weisenhorn P. Gilbert J. A. Shi Y. Bai Y. Chu H. Y. (2018). Soil pH correlates with the co-occurrence and assemblage process of diazotrophic communities in rhizosphere and bulk soils of wheat fields. Soil Biol. Biochem. 121, 185–192. doi: 10.1016/j.soilbio.2018.03.017

[ref15] George L. K. Edith B. L. (2005). Apical GLUT2: a major pathway of intestinal sugar absorption. Diabetes 54, 3056–3062. doi: 10.2337/diabetes.54.10.3056, PMID: 16186415

[ref16] Ghosh N. K. Cox R. P. (1977). Induction of human follicle-stimulating hormone in HeLa cells by sodium butyrate. Nature 267, 435–437. doi: 10.1038/267435a0, PMID: 876359

[ref17] Gomathi G. Senthilkumar S. Natarajan A. Amurtha R. Purushothaman M. R. (2018). Effect of dietary supplementation of cinnamon oil and sodium butyrate on carcass characteristics and meat quality of broiler chicken. Vet. World 11, 959–964. doi: 10.14202/vetworld.2018.959-964, PMID: 30147266 PMC6097553

[ref18] Gong H. Lang W. Lan H. Fan Y. Wang T. Chu Q.. (2020). Effects of laying breeder hens dietary β-carotene, curcumin, allicin, and sodium butyrate supplementation on the jejunal microbiota and immune response of their offspring chicks. Poult. Sci. 99, 3807–3816. doi: 10.1016/j.psj.2020.03.065, PMID: 32731966 PMC7597918

[ref19] Honma K. Oshima K. Takami S. Goda T. (2020). Regulation of hepatic genes related to lipid metabolism and antioxidant enzymes by sodium butyrate supplementation. Metab. Open 7:100043. doi: 10.1016/j.metop.2020.100043, PMID: 32812944 PMC7424775

[ref20] Humphrey B. D. Klasing K. C. (2004). Modulation of nutrient metabolism and homeostasis by the immune system. World Poult. Sci. J. 60, 90–100. doi: 10.1079/WPS20037

[ref21] Jahanian R. Golshadi M. (2015). Effect of dietary supplementation of butyric acid glycerides on performance, immunological responses, ileal microflora, and nutrient digestibility in laying hens fed different basal diets. Livest. Sci. 178, 228–236. doi: 10.1016/j.livsci.2015.05.038

[ref22] Jiang Y. Zhang W. Gao F. Zhou G. (2015). Effect of sodium butyrate on intestinal inflammatory response to lipopolysaccharide in broiler chickens. Can. J. Anim. Sci. 95, 389–395. doi: 10.4141/cjas-2014-183

[ref23] Jiao S. Liu Z. S. Lin Y. B. Yang J. Chen W. M. Wei G. H. (2016). Bacterial communities in oil contaminated soils: biogeography and co-occurrence patterns. Soil Biol. Biochem. 9, 64–73. doi: 10.1016/j.soilbio.2016.04.005

[ref24] Kannan K. Jain S. K. (2000). Oxidative stress and apoptosis. Pathophysiology 7, 153–163. doi: 10.1016/S0928-4680(00)00053-5, PMID: 10996508

[ref25] Lee K. C. Kil D. Y. Sul W. J. (2017). Cecal microbiome divergence of broiler chickens by sex and body weight. J. Microbiol. 55, 939–945. doi: 10.1007/s12275-017-7202-0, PMID: 29214491

[ref26] Li J. Tan B. Mai K. (2009). Dietary probiotic *Bacillus* OJ and isomaltooligosaccharides influence the intestine microbial populations, immune responses and resistance to white spot syndrome virus in shrimp (*Litopenaeus vannamei*). Aquaculture 291, 35–40. doi: 10.1016/j.aquaculture.2009.03.005

[ref27] Liao X. Wu R. Ma G. Zhao L. Zheng Z. Zhang R. (2015). Effects of *Clostridium butyricum* on antioxidant properties, meat quality and fatty acid composition of broiler birds. Lipids Health Dis. 14:36. doi: 10.1186/s12944-015-0035-0, PMID: 25896790 PMC4409984

[ref28] Liu M. Guo W. Wu F. Qu Q. Tan Q. Gong W. (2017). Dietary supplementation of sodium butyrate may benefit growth performance and intestinal function in juvenile grass carp (*Ctenopharyngodon idellus*). Aquac. Res. 48, 4102–4111. doi: 10.1111/are.13230

[ref29] Makled M. N. Abouelezz K. F. M. Gad-Elkareem A. E. G. Sayed A. M. (2019). Comparative influence of dietary probiotic, yoghurt, and sodium butyrate on growth performance, intestinal microbiota, blood hematology, and immune response of meat-type chickens. Trop. Anim. Health Prod. 51, 2333–2342. doi: 10.1007/s11250-019-01945-8, PMID: 31168683

[ref30] Mangian H. F. Tappenden K. A. (2009). Butyrate increases GLUT2 mRNA abundance by initiating transcription in Caco2-BBe cells. J. Parenter. Enteral. Nutr. 33, 607–617. doi: 10.1177/0148607109336599, PMID: 19892901

[ref31] Meimandipour A. Shuhaimi M. Soleimani A. F. Azhar K. S. Hair-Bejo M. Kabeir B. M.. (2010). Selected microbial groups and short-chain fatty acids profile in a simulated chicken cecum supplemented with two strains of *Lactobacillus*. Poult. Sci. 89, 470–476. doi: 10.3382/ps.2009-00495, PMID: 20181862

[ref32] Nadia R. Hassan R. A. Qota E. M. Fayek H. M. (2008). Effect of natural antioxidant on oxidative stability of eggs and productive and reproductive performance of laying hens. Int. J. Poult. Sci. 7, 134–150. doi: 10.3923/ijps.2008.134.150

[ref33] Satoh T. Takeuchi O. Vandenbon A. Yasuda K. Kumagai Y. Miyake T.. (2010). The Jmjd3-Irf4 axis regulates M2 macrophage polarization and host responses against helminth infection. Nat. Immunol. 11, 936–944. doi: 10.1038/ni.1920, PMID: 20729857

[ref34] Sauer J. Richter K. K. Pool-Zobel B. L. (2007). Physiological concentrations of butyrate favorably modulate genes of oxidative and metabolic stress in primary human colon cells. J. Nutr. Biochem. 18, 736–745. doi: 10.1016/j.jnutbio.2006.12.012, PMID: 17434725

[ref35] Scanes C. G. ed. (2015). Sturkie’s Avian Physiology, 6th *Edn*. London, UK: Academic Press.

[ref36] Shimizu T. Kawasaki Y. Aoki Y. Magata F. Kawashima C. Miyamoto A. (2017). Effect of single nucleotide polymorphisms of toll-like receptor 4 (TLR 4) on reproductive performance and immune function in dairy cows. Biochem. Genet. 55, 212–222. doi: 10.1007/s10528-017-9790-0, PMID: 28093679

[ref37] Sikandar A. Zaneb H. Younus M. Masood S. Aslam A. Khattak F.. (2017). Effect of sodium butyrate on performance, immune status, microarchitecture of small intestinal mucosa and lymphoid organs in broiler chickens. Asian Australas. J. Anim. Sci. 30, 690–699. doi: 10.5713/ajas.16.0824, PMID: 28111438 PMC5411829

[ref38] Soltan M. A. (2008). Effect of dietary organic acid supplementation on egg production, egg quality and some blood serum parameters in laying hens. Int. J. Poult. Sci. 7, 613–621. doi: 10.3923/ijps.2008.613.621

[ref40] Subramanya S. B. Rajendran V. M. Srinivasan P. Nanda Kumar N. S. Binder H. J. (2007). Differential regulation of cholera toxin-inhibited Na-H exchange isoforms by butyrate in rat ileum. Am. J. Physiol. Gastrointest. Liver Physiol. 293, G857–G863. doi: 10.1152/ajpgi.00462.2006, PMID: 17690171

[ref41] Sunkara L. T. Achanta M. Schreiber N. B. Bomminein Y. R. Dai G. Jiang W.. (2011). Butyrate enhances disease resistance of chickens by inducing antimicrobial host defense peptide gene expression. PLoS One 6:e27225. doi: 10.1371/journal.pone.0027225, PMID: 22073293 PMC3208584

[ref42] Taherpour K. Moravej H. Shivazad M. Adibmoradi M. Yakhchali B. (2009). Effects of dietary probiotic, prebiotic and butyric acid glycerides on performance and serum composition in broiler chickens. Afr. J. Biotechnol. 8, 2329–2334.

[ref43] Takahashi M. Mccartney E. Knox A. Francesch M. Oka K. Wada K.. (2018). Effects of the butyric acid-producing strain *Clostridium butyricum* MIYAIRI 588 on broiler and piglet zootechnical performance and prevention of necrotic enteritis. Anim. Sci. J. 89, 895–905. doi: 10.1111/asj.13006, PMID: 29603498

[ref44] Tappenden K. A. McBurney M. I. (1998). Systemic short-chain fatty acids rapidly alter gastrointestinal structure, function, and expression of early response genes. Dig. Dis. Sci. 43, 1526–1536. doi: 10.1023/A:1018819032620, PMID: 9690391

[ref45] Tappenden K. A. Thomson A. B. Wild G. E. McBurney M. I. (1997). Short chain fatty acid-supplemented total parenteral nutrition enhances functional adaptation to intestinal resection in rats. Gastroenterology 112, 792–802. doi: 10.1053/gast.1997.v112.pm9041241, PMID: 9041241

[ref47] van der Houwen T. B. van Laar J. A. M. Kappen J. H. van Hagen P. M. de Zoete M. R. van Muijlwijk G. H.. (2020). Behçet’s disease under microbiotic surveillance? A combined analysis of two cohorts of Behçet’s disease patients. Front. Immunol. 11:1192. doi: 10.3389/fimmu.2020.01192, PMID: 32595645 PMC7303268

[ref48] Van Deun K. Haesebrouck F. Van Immerseel F. Ducatelle R. Pasmans F. (2008). Short chain fatty acids and L-lactate as feed additives to control *Campylobacter jejuni* infections in broilers. Avian Pathol. 37, 379–383. doi: 10.1080/03079450802216603, PMID: 18622853

[ref49] Wang W. Wang J. Zhang H. Wu S. Qi G. (2020). Effects of *Clostridium butyricum* on production performance and intestinal absorption function of laying hens in the late phase of production. Anim. Feed Sci. Technol. 264:114476. doi: 10.1016/j.anifeedsci.2020.114476

[ref50] Xia W. G. Chen W. Abouelezz K. Ruan D. Mohammed K. (2020). The effects of dietary Se on productive and reproductive performance, tibial quality, and antioxidant capacity in laying duck breeders. Poult. Sci. 99, 3971–3978. doi: 10.1016/j.psj.2020.04.006, PMID: 32731984 PMC7597912

[ref51] Xiao S. Jiang S. Qian D. Duan J. (2020). Modulation of microbially derived short-chain fatty acids on intestinal homeostasis, metabolism, and neuropsychiatric disorder. Appl. Microbiol. Biotechnol. 104, 589–601. doi: 10.1007/s00253-019-10312-4, PMID: 31865438

[ref52] Xing X. Jiang Z. Tang X. Wang P. Li Y. Sun Y.. (2016). Sodium butyrate protects against oxidative stress in HepG2 cells through modulating Nrf2 pathway and mitochondrial function. J. Physiol. Biochem. 73, 405–414. doi: 10.1007/s13105-017-0568-y, PMID: 28600747

[ref53] Yang C. Cao G. Ferket P. R. Liu T. Zhou L. Zhang L.. (2012). Effects of probiotic, *Clostridium butyricum*, on growth performance, immune function, and cecal microflora in broiler chickens. Poult. Sci. 91, 2121–2129. doi: 10.3382/ps.2011-02131, PMID: 22912445

[ref54] Zhan H. Dong X. Li L. Zheng Y. Gong Y. Zou X. (2019). Effects of dietary supplementation with *Clostridium butyricum* on laying performance, egg quality, serum parameters, and cecal microflora of laying hens in the late phase of production. Poult. Sci. 98, 896–903. doi: 10.3382/ps/pey436, PMID: 30285187

[ref55] Zhang B. Yang X. Guo Y. Long F. (2011). Effects of dietary lipids and *Clostridium butyricum* on the performance and the digestive tract of broiler chickens. Arch. Anim. Nutr. 65, 329–339. doi: 10.1080/1745039X.2011.568274, PMID: 21888038

[ref56] Zhang L. Zhang L. Zhan X. Zeng X. Zhou L. Gao G.. (2016). Effects of dietary supplementation of probiotic, *Clostridium butyricum*, on growth performance, immune response, intestinal barrier function, and digestive enzyme activity in broiler chickens challenged with *Escherichia coli* K88. J. Anim. Sci. Biotechnol. 7:3. doi: 10.1186/s40104-016-0061-4, PMID: 26819705 PMC4728939

[ref57] Zhang D. Zhou C. Fang Z. Zhang H. Yang J. Tao K.. (2020). Remodeling gut microbiota by *Clostridium butyricum* (*C. butyricum*) attenuates intestinal injury in burned mice. Burns 46, 1373–1380. doi: 10.1016/j.burns.2020.01.007, PMID: 32014349

[ref58] Zheng X. T. Duan Y. F. Dong H. B. Zhang J. S. (2017). Effects of dietary *Lactobacillus plantarum* in different treatments on growth performance and immune gene expression of white shrimp *Litopenaeus vannamei* under normal condition and stress of acute low salinity. Fish Shellfish Immunol. 62, 195–201. doi: 10.1016/j.fsi.2017.01.015, PMID: 28108342

[ref59] Zhou Z. Y. Packialakshmi B. Makkar S. K. Dridi S. Rath N. C. (2014). Effect of butyrate on immune response of a chicken macrophage cell line. Vet. Immunol. Immunopathol. 162, 24–32. doi: 10.1016/j.vetimm.2014.09.002, PMID: 25278494

[ref60] Zhou Y. Zhi F. (2016). Lower level of Bacteroides in the gut microbiota is associated with inflammatory bowel disease: a meta-analysis. Biomed Res. Int. 2016:5828959. doi: 10.1155/2016/5828959, PMID: 27999802 PMC5143693

[ref61] Zilber-Rosenberg I. Rosenberg E. (2008). Role of microorganisms in the evolution of animals and plants: the hologenome theory of evolution. FEMS Microbiol. Rev. 32, 723–735. doi: 10.1111/j.1574-6976.2008.00123.x, PMID: 18549407

