# Supplementary material for: Effects of Clostridium butyricum, Sodium Butyrate, and Butyric Acid Glycerides on the Reproductive Performance, Egg Quality, Intestinal Health, and Offspring Performance of Yellow-Feathered Breeder Hens
Source: Front Microbiol. 2021 Sep 16;12:657542. doi: 10.3389/fmicb.2021.657542 (PMC8481923; doi:10.3389/fmicb.2021.657542)
Supplement: Supplementary file 1 [file Data_Sheet_1.docx]

**Table S1 Composition and nutrient levels of the basal diet for broiler breeder hens (air-dry basis)**

| **Items** |  | |
| --- | --- | --- |
| Ingredients, % |  | |
| Corn | 63.30 | |
| Soybean meal | 19.00 | |
| Wheat bran | 8.00 | |
| *DL*-Met | 0.30 | |
| *L*-lys（78%） | 0.05 | |
| Limestone | 6.50 | |
| CaHPO_4_ | 1.60 | |
| NaCl | 0.25 | |
| Premix^1)^ | 1.00 | |
| Total | 100.00 | |
| Nutrient Levels^2）^ | |  |
| ME /（kcal/kg） | 2650 | |
| CP | 15.50 | |
| Ca | 2.80 | |
| Lys | 0.38 | |
| Met+Cys | 0.80 | |

^1)^ The premix provided the following per kg of diets: VA 15 000 IU, VD 3 600 IU, VE 47 IU, VK 6 mg, VB_1_ 3 mg, VB_2_ 9 mg, VB_6_ 6 mg, VB_12_ 0.03 mg, niacin 60 mg, *D*-pantothenic acid 16 mg, folic acid 1.5 mg, biotin 0.06 mg, choline 900 mg, Cu, 7.0 mg; Zn, 72 mg; Mn, 90 mg; Fe, 72 mg; I, 0.90 mg; Se, 0.27 mg.

^2）^Nutrient levels were all calculated values.

^3）^Met = methionine, Lys = lysine, Cys = cysteine, ME = metabolizable energy, CP = crude protein, Ca = calcium.

**Table S2 Composition and nutrient levels of the basal diet (air-dry basis) for offspring broilers**

| **Items** | 1 to 21 d | | 22 to 42 d | | 43 to 63 d | |  |
| --- | --- | --- | --- | --- | --- | --- | --- |
| Ingredients, % |  | |  | |  | |  |
| Corn | 61.93 | | 64.40 | | 68.40 | |  |
| Soybean meal | 28.40 | | 23.60 | | 16.15 | |  |
| Corn gluten meal | 2.00 | | 3.00 | | 4.00 | |  |
| Fish meal | 2.00 | | 1.00 | | 0.00 | |  |
| Soybean oil | 1.00 | | 2.50 | | 4.00 | |  |
| Limestone | 1.20 | | 1.10 | | 1.04 | |  |
| Ca(H_2_PO_4_)_2_ | 1.70 | | 1.60 | | 1.55 | |  |
| NaCl | 0.09 | | 0.10 | | 0.11 | |  |
| *DL*-Met | 0.18 | | 0.11 | | 0.13 | |  |
| *L*-LysHCl (78%) | 0.00 | | 0.10 | | 0.25 | |  |
| Ile (84%) | 0.00 | | 0.00 | | 0.02 | |  |
| Thr (85%) | 0.00 | | 0.01 | | 0.10 | |  |
| Zeolite | 0.50 | | 1.48 | | 3.25 | |  |
| Vitamin and mineral premix^1)^ | 1.00 | | 1.00 | | 1.00 | |  |
| Total | 100.00 | | 100.00 | | 100.00 | |  |
| Nutrient Levels^2）^ | |  | |  | |  | |
| ME /(Kcal/Kg) | 2900 | | 3000 | | 3100 | |  |
| CP, % | 21.00 | | 19.00 | | 16.00 | |  |
| Ca, % | 1.00 | | 0.90 | | 0.81 | |  |
| Lys, % | 0.45 | | 0.40 | | 0.36 | |  |
| Met, % | 1.07 | | 0.98 | | 0.86 | |  |
| Met+Cys, % | 0.52 | | 0.42 | | 0.40 | |  |

^1)^ Premix provided the following per kilogram of diets during 1 to 21 days of age: VA 15 000 IU, VD_3_ 3 300 IU, VE 20 IU, VK_3_ 6 mg, VB_1_ 1.8 mg, VB_2_ 9 mg, VB_6_ 3.5 mg, VB_12_ 0.01 mg, 500 mg, 60 mg, 16 mg, 0.55 mg, 0.15 mg, Fe 80 mg, Cu 8 mg, Zn 60 mg, Mn 80 mg, I 0.35 mg, Se 0.3 mg. Premix provided the following per kilogram of diets during 22 to 42 days of age: VA 15 000 IU, VD_3_ 3 300 IU, VE 20 IU, VK_3_ 6.0 mg, VB_1_ 3.0 mg, VB_2_ 9.0 mg, VB_6_ 6.0 mg, VB_12_ 0.03 mg, 1 000 mg, 60 mg, 18 mg, 0.75 mg, 0.10 mg, Fe 80 mg, Cu 12 mg, Zn 75 mg, Mn 60 mg, I 0.35 mg , Se 0.15 mg. Premix provided the following per kilogram of diets during 43 to 63 days of age: VA 10 000 IU, VD_3_ 1 000 IU, VE 20 IU, VK_3_ 4 mg, VB_1_ 1.8 mg, VB_2_ 8 mg, VB_6_ 3.5 mg, VB_12_ 0.01 mg, 500 mg, 44 mg, 10 mg, 0.55 mg, 0.15 mg, Fe 80 mg, Cu 8 mg, Zn 60 mg, Mn 80 mg, I 0.35 mg , Se 0.15 mg

^2）^Nutrient levels were all calculated values.

^3）^Met = methionine, Lys = lysine, Ile = isoleucine, Thr = threonine, Cys = cysteine, ME = metabolizable energy, CP = crude protein, Ca = calcium.

**Table S3 PCR primer sequences**

| Gene | Primers (5’-3’) | Annealing temperature (℃) | |
| --- | --- | --- | --- |
| *NHE2* | F: GCAGATCCCCTTCGAGATCA  R: CCAGCGTCGAGTACAATTGG | | 57 |
| *PEPT1* | F: TAGACTGGGCAAGCGAGAAG  R: AGCAGCAGCAACGAAAGC | | 60 |
| *GLUT2* | F: GGTGGTCAATGTCCTCTCCA  R: CACTCACATACATGGGCACG | | 60 |
| *B0AT* | F: TCTGCCTGGGTTTGTCATCT  R: AGCCAGTAATTGCCAGACCT | | 60 |
| *TNF-α* | F: GAAGCAGCGTTTGGGAGTG  R: GTTGTGGGACAGGGTAGGG | | 56 |
| *BAX* | F: GTGATGGCATGGGACATAGCTC  R: TGGCGTAGACCTTGCGGATAA | | 58 |
| *β -actin* | F: GAGAAATTGTGCGTGACATCA  R: CCTGAACCTCTCATTGCCA | | 60 |

NHE2 = Na(+)/H(+) exchanger isoform 2, PEPT1 = peptide transporter 1, GLUT 2 = glucose transporter-2, B^0^AT = [apical nutrient transporter SLC6A19](https://www.jbc.org/content/288/47/33813.short), TNF-α = tumor necrosis factor α, BAX = Bcl-2 associated X protein.
